# Elevated levels of circulating IL-18BP and perturbed regulation of IL-18 in schizophrenia

**DOI:** 10.1186/1742-2094-9-206

**Published:** 2012-08-22

**Authors:** Ilaria Palladino, Francesca Salani, Antonio Ciaramella, Ivo Alex Rubino, Carlo Caltagirone, Sabrina Fagioli, Gianfranco Spalletta, Paola Bossù

**Affiliations:** 1 Clinical and Behavioral Neurology, IRCCS Fondazione Santa Lucia, Via Ardeatina 306, 00179, Rome, Italy; 2 Department of Neuroscience, University Tor Vergata, Via Montpellier 1, 00133, Rome, Italy; 3 Department of Clinical and Behavioral Neurology, Experimental Neuro-psychobiology Lab, IRCCS Santa Lucia Foundation, Via Ardeatina, 306, I-00179, Rome, Italy

**Keywords:** Interleukin-18, Interleukin-18 Binding Protein, Inflammation, Schizophrenia

## Abstract

**Background:**

The pleiotropic pro-inflammatory cytokine Interleukin (IL)-18 has been proposed to play a role in schizophrenia, since elevated circulating levels of its protein and altered frequencies of genetic variants in its molecular system are reported in schizophrenic patients.

**Methods:**

We analyzed 77 patients with schizophrenia diagnosis (SCZ) and 77 healthy control subjects (HC) for serum concentration of both IL-18 and its natural inhibitor, the IL-18 binding protein (IL-18BP).

**Results:**

We confirmed that serum levels of total IL-18 are significantly increased in SCZ, as compared to HC. However, due to a highly significant increase in levels of circulating IL-18BP in SCZ, as compared to HC, the levels of free, bioactive IL-18 are not significantly different between the two groups. In addition, the relationships between the levels of IL-18 and its inhibitor, as well as between the two molecules and age appear dissimilar for SCZ and HC. In particular, the elevated levels of IL-18BP, likely a consequence of the body’s attempt to counteract the early prominent inflammation which characterizes schizophrenia, are maintained in earlier and later stages of the disease. However, the IL-18BP elevation appears ineffective to balance the IL-18 system in younger SCZ patients, while in older patients the levels of circulating bioactive IL-18 are comparable to those of HC, if not lower.

**Conclusions:**

In conclusion, these findings indicate that the IL-18 system is perturbed in schizophrenia, supporting the idea that this pro-inflammatory cytokine might be part of a pathway of genetic and environmental components for vulnerability to the disease.

## Background

Interleukin (IL)-18 is a pleiotropic pro-inflammatory molecule acting as a potent promoter of chronic inflammation, autoimmunity and age-dependent inflammatory processes [1,2]. Recently, IL-18 has been found to play a diverse array of functions in non-immune tissues as the central nervous system [3], and its abnormal expression has been observed both centrally and peripherally in neuropsychiatric disorders such as Alzheimer’s disease and stroke [4,5].

The bioactivity of IL-18 is modulated by a secreted binding protein (IL-18BP) with neutralizing abilities, which binds IL-18 with high affinity (*K*_d_ = 0.4 nM) and prevents its interaction with the receptor [6,7]. IL-18BP is constitutively present in the serum of healthy humans at molar excess when compared with IL-18 [8], and it appears to be modulated during the life. In fact, since IL-18 participates in fundamental inflammatory processes that increase during the aging process [2] and IL-18BP serum levels tend to increase in old age with evidence of strong increment in healthy centenarians [9], it has been suggested that IL-18BP retains a remarkable protective role by acting to limit the impact of age-related inflammation. In addition, IL-18BP appears to be strongly modulated in pathological circumstances, as it increases with the increase of IL-18 production in inflammatory conditions [10], likely operating a negative feedback mechanism that is able to reduce IL-18-elicited immune responses [11].

Schizophrenia is a familiar multigenic disorder with probable neurodevelopmental origins, characterized by a wide range of psychotic symptoms that appear in adolescence and persist throughout the life of affected individuals. Among the several possible hypotheses formulated to explain the genesis of schizophrenia, immune system vulnerability is one of the most recognized [12,13]. A possible link between immune function changes and schizophrenia development is the stimulation of cytokine response. This has prompted renewed interest in cytokines as markers of immune imbalance in schizophrenia and cytokine-centered hypotheses of schizophrenia pathogenesis have been recently proposed [14]. In agreement with the idea that imbalances of pro- and anti-inflammatory cytokines may contribute to the onset of psychotic symptoms and the progressive loss of brain tissue in schizophrenia, IL-18 has been proposed to be connected to this disorder. In particular, five polymorphisms in genes related to the IL-18 pathway, including IL-18 receptor genes, have been associated with schizophrenia [15] and some variants of the *IL-18* gene have been recently related to the development of schizophrenia symptoms [16]. Furthermore, levels of IL-18 circulating protein are higher in affected patients than in controls [17,18] and are associated with the psychopathology of schizophrenia [19].

However, the IL-18 biology in schizophrenia is still unclear and no data are available on the relationship between IL-18 and IL-18BP in affected individuals. Therefore, in the present study we analyzed IL-18 and IL-18BP peripheral levels in subjects with schizophrenia diagnosis (SCZ) in comparison to healthy control subjects (HC).

## Materials and methods

### Subjects

Seventy-seven SCZ diagnosed according to the Diagnostic and Statistical Manual of Mental Disorders-IV edition (DSM-IV) as previously reported [20] were recruited from outpatient clinics in Central Italy. Seventy-seven healthy control subjects (HC) were recruited in the same geographic area and matched with the SCZ for age and gender, as summarized in Table 1. Exclusion criteria were: 1) treatment with anti-inflammatory or immunosuppressive medication; 2) overt infectious disease or auto-immune disease; 3) history of alcohol or drug dependence or traumatic head injury; 4) any past or present major medical or neurological illness. This research was carried out with the ethical approval of Santa Lucia Foundation institutional review board. Written informed consent was obtained from each subject after giving subjects a complete description of the study.

**Table 1:** Clinical and biological characteristics of schizophrenic and healthy control subjects

** *Characteristics* **	** *Healthy control subjects (HC, n = 77)* **	** *Schizophrenic patients (SCZ, n = 77)* **
Males/Females (n)	49/28	49/28
Age (years ± SEM)	41.8 ± 1.4	40.9 ± 1.3^*a*^
Serum IL-18 (pg/ml ± SEM)	382 ± 16.5	518.2 ± 22.1^b^
Serum IL-18BP (ng/ml ± SEM)	4.9 ± 0.2	9.8 ± 0.4^b^
Serum free IL-18 (pg/ml ± SEM)	239.3 ± 9.8	253.6 ± 12.1^*a*^
Disease onset age (years ± SEM)	-	25.4 ± 1.1
Duration of illness (years ± SEM)	-	14.9 ± 1.6
Olanzapine equivalents (mg/day ± SEM)	-	22.3 ± 5.5

^*a*^*difference is not significant versus HC;*^*b*^*P <0.0001 versus HC.*

All patients were receiving stable oral doses of one or more atypical antipsychotic drugs such as risperidone, quetiapine, and olanzapine. Antipsychotic dosages were converted to estimated equivalent dosages of olanzapine.

### Serum and IL-18/IL-18BP measurement

Serum samples were obtained from all subjects by centrifugation of clotted blood, and aliquots were stored at −80°C until cytokine assays were performed.

Total IL-18, corresponding to the total amount of free IL-18 and IL-18 bound to IL-18BP, was determined by enzyme-linked immunosorbent assay (ELISA), using antibodies unable to distinguish between the free and the bound form of IL-18. More in detail, coating antibody (clone 125–2 H), detecting antibody (clone 159-12B) and standard human recombinant IL-18, were used (MBL, Nagoya, Japan). IL-18BP was measured using commercial ELISA (R&D Systems Minneapolis, MN, USA) specific for IL-18BPa, the prevalent isoform in humans, in accordance with the manufacturer’s instructions. The limit of detection for both assays was 12.5 pg/mL. Concentrations of free IL-18, namely unbound to IL-18BP, resulted from calculation based on the law of mass action, considering 1:1 stoichiometry in the complex of IL-18 and IL-18BP and a dissociation constant (K*d*) of 0.4 nM, as elsewhere reported in more detail [10,21].

### Statistical analysis

In order to detect statistically significant differences, comparisons between continuous variables were made using the Student’s *t* test. Pearson’s correlation coefficient was used to assess the relationships between continuous variables. The level of statistical significance was defined as *P <*0.05. GraphPad (Prism Version 4, San Diego, CA) software was used for statistical analyses.

## Results

### Serum levels of IL-18/IL-18BP

**Figure 1:**
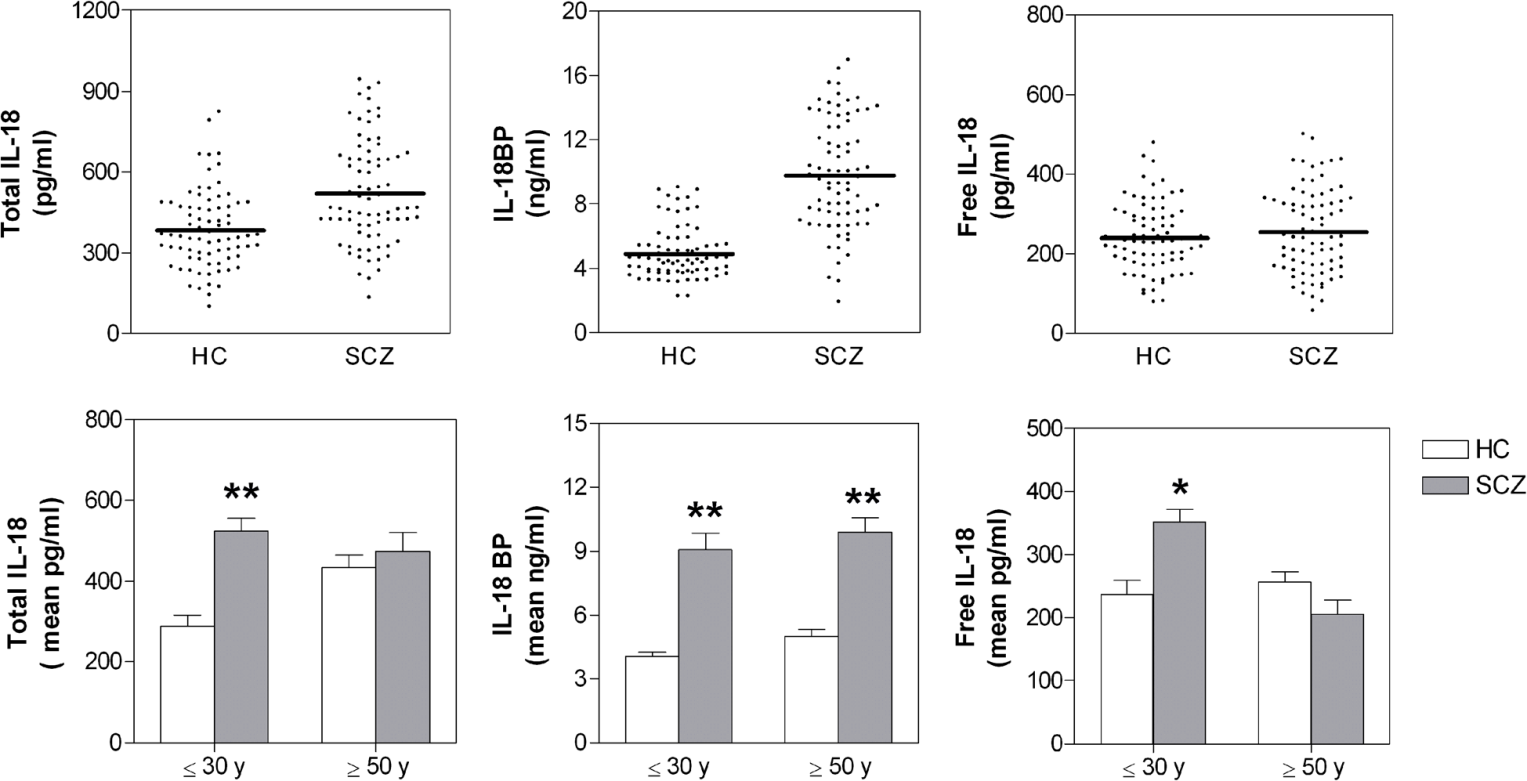
**Concentration of IL-18, IL-18BP and free IL-18 in serum of all HC (n = 77) and SCZ (n = 77) subjects (upper panels) and in two age subgroups of HC and SCZ subjects (lower panels), including subjects with age ≤ 30 years (HC: n = 15, mean age = 24.4 ± 0.7; SCZ: n = 18, mean age = 25.6 ± 0.7) and age ≥ 50 years (HC: n = 22, mean age = 56.5 ± 1; SCZ: n = 19, mean age = 56.1 ± 1.1).** Asterisks inside the graphs indicate the significance of comparisons of SCZ *vs*. HC: * *P* < .001, ** *P <* .0001. HC, healthy controls; SCZ, schizophrenic patients.

The concentration of both IL-18 and IL-18BP was evaluated for the serum of each subject. In order to define the levels of bioactive cytokine, free IL-18 was also calculated on the basis of IL-18 and IL-18BP results. Mean values of total or unbound IL-18 and its inhibitor concentration are reported in Table 1, while individual values are reported in the upper panels of Figure 1. Total IL-18 levels were significantly higher in SCZ than HC (*P<*0.0001). Similarly, serum levels of IL-18BP were significantly higher in SCZ than HC (*P*<0.0001). Interestingly, serum levels of free, bioactive IL-18 were not significantly different between SCZ and HC. The levels of the three elements of IL-18 system were not significantly different between males and females and did not correlate significantly with illness duration and antipsychotic drug dosages.

### Relationship between IL-18, IL-18BP and age

In order to evaluate a possible relationship between total IL-18 and IL-18BP levels, we first assessed the relationship existing between the two immune mediators both in SCZ and HC. A positive significant correlation was found in SCZ (*r =* 0.245, *P =* 0.03) but not in HC.

Afterwards, given that IL-18 might participate in age-dependent inflammation, we analyzed the direct relationship between the serum concentration of total IL-18, IL-18BP, free IL-18 and age in all subjects. Indeed, a significant positive correlation between serum total IL-18 levels and age was found in HC (*r =* 0.268; *P =* 0.018), but not in SCZ. No significant correlations were observed between the levels of IL-18BP and age in both HC and SCZ. Differently, an unexpected and highly significant inverse correlation was found between the serum amount of free IL-18 and age in SCZ (*r =* −0.432; *P <*0.0001) but not in HC.

In order to further investigate the age dependence of the IL-18 system, we compared the changes in the levels of IL-18-related molecules in HC and SCZ after dividing each main group into two subgroups formed by the youngest (≤ 30 years; HC, n = 15 and SCZ, n = 18) and the oldest (≥50 years; HC, n = 22 and SCZ, n = 19) subjects. As shown in the lower panels of Figure 1, SCZ showed an increase of total IL-18, as compared to HC, in the youngest (1.82-fold increase; *P*<0.0001) but not in the oldest individuals. The levels of IL-18BP were significantly higher in SCZ, as compared to HC, both in the youngest and the oldest subjects, although the increase was slightly higher in the youngest (2.2-fold increase; *P*<0.0001) than the oldest (1.96-fold increase; *P*<0.0001) subgroup. In contrast, the levels of free IL-18 were significantly higher in SCZ than HC as for the subgroup of the youngest subjects (1.48-fold increase; *P* = 0.0006), while the biologically active cytokine has a tendency to decrease in SCZ *versus* HC in individuals with an age ≥50 years (1.24-fold decrease; *P* = 0.068).

## Discussion

It is known that the pro-inflammatory cytokine IL-18 is present in the circulation partly as an inactive complex with IL-18BP and partly as free, bioactive IL-18 [6,7]. Previous studies indicate that schizophrenia is paralleled by elevated levels of serum IL-18 without any characterization of the active form of the cytokine [17,18]. In the present report we assessed the levels of both IL-18 and IL-18BP in SCZ and age- and gender-matched HC. We confirmed that the total amount of IL-18, including the unbound and the IL-18BP-bound form of the cytokine, is higher in SCZ than in HC. Nevertheless, the main finding of this study is that the cytokine inhibitor IL-18BP is significantly increased in the serum of SCZ, as compared to HC. Interestingly, this elevation is about 40% higher than that of total IL-18. Thus, the evident increase of IL-18BP in SCZ creates a balance in the levels of free active IL-18, which, in fact, appear comparable between patients and HC subjects.

As a further indication of the altered modulation of IL-18 system in schizophrenia, we found a positive correlation between total IL-18 and its inhibitor only in SCZ and not in HC. This correlation may be linked to the fact that SCZ show, in comparison with HC, a greater range of values in IL-18BP levels. This, together with the observation occurring in autoimmune conditions that IL-18BP is triggered when the IL-18 response is amplified [10], leads us to hypothesize that IL-18BP elevation in SCZ is linked to an early and disease-dependent increase of IL-18. Accordingly, when we compared the levels of IL-18 system molecules of SCZ and HC belonging to different age groups, we confirmed the previous assumption that in HC there is a trend of IL-18 to increase with age [2,9]. Conversely, this age-associated modulation of the IL-18 system appears altered in SCZ, thus strengthening the concept of an IL-18 system perturbation associated with the disorder. We established that in SCZ subjects, as compared to age-matched HC, the increase of total and free IL-18 was observable only in the youngest individuals, where the elevation of IL-18BP did not seem to reach adequate levels to reduce free IL-18 to HC levels, thus reflecting an imbalance which might favor a general inflammatory state in the early phase of the disorder. Noteworthy, in agreement with the condition we have described for young SCZ patients, a number of studies has previously reported disease-related high levels of free IL-18, despite the concomitant over expression of IL-18BP [10,22,23], which is in accordance with the nature of the interaction between IL-18 and IL-18BP [24].

At variance with young individuals, SCZ subjects with an age ≥50 years did not show levels of total IL-18 significantly different from HC, likely a result of the age-dependent increase of total IL-18 values occurring only in HC, which would progressively bring the cytokine levels of the latter to almost reach those of SCZ. Furthermore, in older SCZ the highly significant elevation of IL-18BP persisted and free IL-18 levels appeared slightly lower than those of HC. In this view, a possible modulation of IL-18 system might be envisaged according to the following scenario. In young SCZ, total and free IL-18 increase over normal levels, similar to what has been observed for other pro-inflammatory cytokines, perhaps as a consequence of an inflammatory challenge at the maternal-fetal interface [14]. Therefore, IL-18BP elevation might be interpreted as an effort of the organism to compensate the exaggerated inflammatory response linked to the disorder. This counteraction might become effective in balancing the IL-18-mediated pro-inflammatory reaction only in the later stages.

In agreement with our findings, other recent studies suggest that SCZ subjects are not characterized by a simple pro-inflammatory profile, but more likely the illness is accompanied by both pro- and anti-inflammatory activated forces, which may involve both monocyte and T-cell networks, probably as a product of an inflammatory system control [25,26]. Yet, the sense of this perturbation in IL-18 system, characterized by elevated levels of IL-18BP here described for the first time in the context of schizophrenia, should be deciphered on a time-dependent basis, in the light of its ability to limit harmful inflammation on the one hand and to contribute to increased susceptibility to infection and immune-related diseases, on the other hand [27]. The early increase in free IL-18, which fades away with age in SCZ, consistently points to the importance of considering age and duration of the illness when discussing the immune-related pathophysiological mechanisms of schizophrenia. Longitudinal studies that address the kinetic of IL-18 system players in relation to disease progression can further clarify the role of free IL-18 in SCZ patients.

The interpretation of our results should take into consideration some limitations of the present study. We used a naturalistic approach where all SCZ were receiving psychotropic drugs, though antipsychotic medication might be a confounding factor in the analysis of the immune system in schizophrenia. Nonetheless, in this study there was not a relationship between antipsychotic treatment dosages and the cytokine parameters, suggesting that in our conditions treatment and serum IL-18 system changes might be independent. This is consistent with the findings of two other recent studies which report that the antipsychotic treatment does not significantly modulate the levels of IL-18 in the serum of schizophrenic patients [18,19], even though the cytokine produced by patients’ blood cells is actually increased by therapy [18]. In conclusion, regardless of whether the IL-18 dysregulation is of genetic, infectious or other origin, our findings strongly suggest that the elevation of IL-18BP found in our SCZ, as well as the whole perturbation of the IL-18 system, might be indeed a specific phenomenon of schizophrenia. Given the hypothesis of immune dysfunction in this illness and the emergent interest in inflammatory mechanisms as potential targets for its treatment [27,28], our results may offer new insights into the field. In fact, even though further clarification of IL-18 role in the disease is certainly needed, the described data suggest that more attention should be paid to the analysis of circulating cytokines, which ought to include the contextual measurement of cytokine natural inhibitors and antagonists in order to develop novel and potentially effective anti-inflammatory therapeutic intervention against schizophrenia.

## Abbreviations

DSM-IV, Diagnostic and Statistical Manual of Mental Disorders; ELISA, Enzyme-Linked Immunosorbent Assay; HC, healthy controls; IL, interleukin; IL-18BP, interleukin-18 binding protein; SCZ, schizophrenia.

## Competing interests

The authors declare that they have no competing interests.

## Authors’ contribution

PB, GS and CC designed and coordinated the study. IAR, SF and GS selected patients and performed all clinical evaluations. IP, FS and AC prepared samples and performed cytokine experiments. All authors made substantial contribution to analysis and discussion of the data. IP wrote the initial draft of the manuscript and PB revised it. All authors made contributions in writing and discussing the manuscript. All authors have read and approved its final version.
